# Adolescents as Co-Designers: How Youth Perspectives Can Shape the Foundation for Mental Health Interventions in Northern Ghana

**DOI:** 10.21203/rs.3.rs-6279575/v1

**Published:** 2025-05-08

**Authors:** Claudia L. Leung, Priscilla Kukua Goka, Barnabas Atangongo, Mohammed Mansur Musah Bingle, Ishmael Adu, Abdul Rashid Atchulo, Laud Boateng, Keng-Yen Huang, Neda Laiteerapong, Gbana Limann, Anna Volerman, Peter Mintir Amadu, William Frank Hill Koomson

**Affiliations:** University of Chicago Biological Sciences Division; University for Development Studies School of Medicine; Tamale West Hospital; University for Development Studies School of Medicine; University for Development Studies School of Medicine; Tamale Teaching Hospital; New York University Grossman School of Medicine; New York University Grossman School of Medicine; University of Chicago Biological Sciences Division; Tamale Teaching Hospital; University of Chicago Biological Sciences Division; University for Development Studies School of Medicine; University for Development Studies School of Medicine

**Keywords:** Adolescent mental health, co-design, human-centered design, school-based interventions, low- and middle-income countries

## Abstract

**Background:**

Adolescents in low- and middle-income countries (LMICs) face significant mental health challenges, yet their voices are often underrepresented in intervention design. Co-design approaches, such as human-centered design, offer a promising approach to tailor interventions to specific needs and context; however, this requires careful adaption in LMICs where resources, design experience, and cultural factors impact engagement and efficacy. This study documents how human-centered design was adapted to engage adolescents in co-designing a school-based mental health intervention, highlighting the contextualization of co-design methods to the Ghanaian sociocultural context and the unique participation of youth.

**Methods:**

Guided by the first two phases of human-centered design, we conducted two workshops with 24 students from 12 public senior high schools in Tamale, Ghana. Workshop 1 (Inspiration) explored adolescent perspectives on mental health using structured case-based discussions guided by the Consolidated Framework for Implementation Research (CFIR). Workshop 2 (Ideation) focused on identifying preferred mental health prevention strategies using interactive, choice-based activities. To accommodate cognitive and sociocultural factors, workshops incorporated structured facilitation, visual analogies, peer-driven engagement, and scaffolded decision-making. Qualitative data from discussions, facilitator notes, and artifacts were analyzed thematically.

**Results:**

Workshop 1 (Inspiration) identified key adolescent mental health concerns, including stigma, confidentiality fears, and peer and family influences. Gender-segregated discussions provided insights into culturally-specific challenges, such as substance use norms among boys and gendered expectations limiting girls’ access to support. Workshop 2 (Ideation) led to the prioritization of five school-based prevention strategies: teacher training, mental health curricular integration, mentorship programs, teaching positive thinking and mindfulness, and using entertainment-based methods for mental health education. Adolescents shifted from viewing mental health challenges as individual struggles to recognizing the role of schools and communities in prevention. An adolescent advisory board was formed to sustain youth engagement in intervention refinement.

**Conclusions:**

Contextualized co-design methods can meaningfully engage adolescents in LMICs, leading to culturally grounded and actionable mental health interventions. Structured facilitation enhances the feasibility and authenticity of youth-driven co-design, contributing methodological insights for implementation science in resource-limited settings. This study provides a replicable framework to apply to diverse LMIC contexts and health topics and elevates youth voices in shaping effective, sustainable interventions.

## INTRODUCTION

Adolescents in low- and middle-income countries (LMICs) face a disproportionate burden of mental health challenges.^1,2^ Globally, 10–20% of adolescents experience mental health conditions, with anxiety, depression, and emotional and behavioral disorders being the most prevalent.^3^ Despite this high prevalence, mental health systems in LMICs are often under-resourced, facing shortages of trained professionals, pervasive stigma surrounding mental illness, and inadequate integration of mental health within health and education systems.^4^ Ensuring adolescent voices are included in intervention development is critical to creating solutions that align with their needs, lived experiences, and sociocultural contexts.

Co-design approaches, such as human-centered design, have emerged as promising strategies for intervention development in both high and low-income countries settings.^5^ Unlike traditional top-down approaches, co-design engages stakeholders as active contributors, ranging from consultative methods (where stakeholders provide input) to fully collaborative processes (where stakeholders and researchers iteratively develop and refine interventions).^6^ Co-design has been more commonly applied with adult stakeholders, often bringing together multiple decision makers to co-create solutions that address specific implementation constraints while also fostering stakeholder ownership and buy-in for long-term sustainability.^7^ However, when it comes to mental health and well-being interventions, youth also desire meaningful participatory engagement,^8^ and their involvement leads to more acceptable, feasible, and engaging mental health interventions,^9,10^ making co-design a valuable implementation strategy for adolescent-focused programs.

Most documented youth co-design applications originate from high-income countries, where participatory methods are supported by well-funded health and education systems.^11^ In LMICs, youth co-design has been used to increase cultural relevance and improve intervention development. For example, the FOOTPATHS FOR MAMA intervention in Malawi engaged pregnant adolescents to co-design a mental health educational booklet,^12,13^ while the SAMA project in India engaged students, teachers, and parents in the development of a school-based mental health intervention.^14,15^ However, many existing LMIC studies describe *what* youth contributed to co-design efforts but not *how* their engagement was structured to overcome participation barriers and ensure meaningful engagement.^16–19^

Our prior work has identified multi-level barriers to school-based mental health support based on perspectives from school and community leaders (*manuscript under review, Leung et al*). These findings underscored the need for adolescent engagement to complement stakeholder perspectives and co-develop interventions that are responsive to the needs and experiences of youth. However, while co-design offers a promising avenue for intervention development, its application in LMICs presents distinct challenges. Most adolescents have limited familiarity with participatory methods like brainstorming and abstract thinking exercises, which are more commonly used in high-income settings.^20–22^ Hierarchical power dynamics, resource constraints, and limited availability of skilled co-design facilitators further hinder participatory processes.^23,24^ These challenges are further compounded when working with adolescents, as they must be addressed alongside developmental differences in cognitive processing and varying levels of health literacy.^25^ Despite growing recognition of co-design as a valuable tool for intervention development and implementation research, practical evidence on how to adapt participatory methods to optimize youth engagement remains limited.^26^ This gap hinders efforts to refine, replicate, and scale adolescent involvement in mental health intervention development. Moreover, most studies focus on the early ideation phase, leaving gaps in understanding of how to sustain youth engagement in later stages of prototyping, implementation, and evaluation. Without structured approaches for ongoing adolescent engagement, interventions risk losing alignment with youth priorities over time.

**Figure 1:**
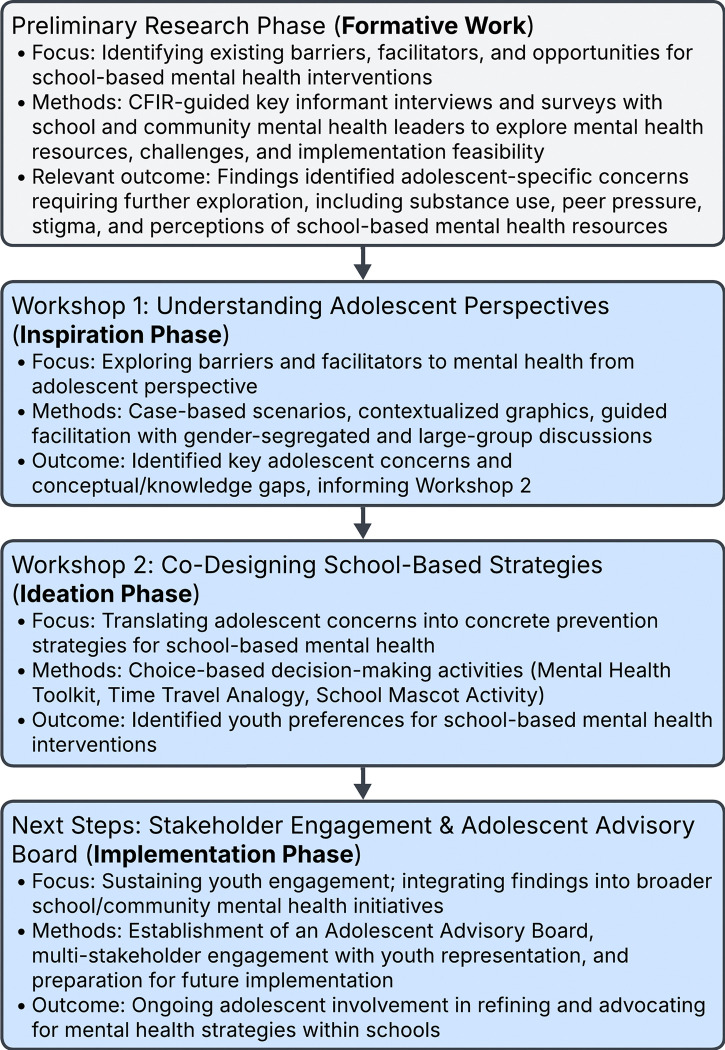
Overview of study phases and co-design activities to develop a school-based mental health intervention. This figure outlines the sequential phases of the study using human-centered design methods in northern Ghana. The process began with a formative research phase that identified key barriers and facilitators to school-based mental health support through key informant interviews and surveys guided by the Consolidated Framework for Implementation Research (CFIR). These findings informed Workshop 1 (Inspiration Phase), which engaged adolescents to explore mental health perceptions, barriers, and facilitators using case-based discussions and contextualized graphics. Workshop 2 (Ideation Phase) guided adolescents in co-designing preferred school-based mental health strategies through interactive choice-based activities. The process culminated in the establishment of an Adolescent Advisory Board to sustain youth engagement during the implementation phase.

This study addresses these gaps by documenting a human-centered design process for a school-based mental health preventative intervention for adolescents in northern Ghana (Fig. 1). Specifically, we aim to (1) document a structured approach for effectively engaging adolescents in intervention development; (2) illustrate context-specific adaptations of co-design processes in the Ghanaian sociocultural setting; and (3) identify adolescent-generated mental health priorities and actionable intervention components. By situating youth co-design within an implementation science framework, this study provides practical insights into contextually tailoring participatory methods in LMICs. Documenting the process, lessons learned, and outcomes offers a replicable model for youth participation in intervention development in resource-limited settings.

## METHODS

### Study setting and population

The study was conducted in Tamale, Ghana, the fastest-growing city in West Africa, characterized by rapid urbanization and social changes that impact adolescent mental health.^27–32^ Public secondary education in Ghana is free and under the purview of Ghana Education Services, which provided access to engage with schools and students in this study. The study population included adolescent representatives from all twelve public senior high schools across the two districts of Tamale: Tamale Metropolis and Sagnarigu. Each school nominated two students (one male, one female, unless it was a single-gender school). Inclusion criteria required that the participants be currently enrolled, provide parental or personal written consent, and be willing and able to participate in discussions in English, Ghana’s official language.

### Study design and data collection

This study employed a human-centered design framework, grounded in IDEO’s three-stage model: Inspiration, Ideation, and Implementation.^33^ The study focused on the first two stages—Inspiration and Ideation—where adolescents were engaged in understanding mental health challenges (inspiration) and co-developing potential solutions (ideation). These phases were selected to ensure that adolescents’ perspectives informed the intervention development process before broader stakeholder engagement in later ideation and implementation phases.

**Figure 2:**
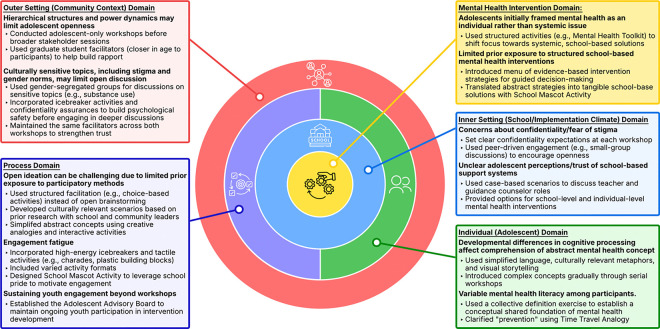
Contextual adaptations to optimize adolescent engagement in co-design workshops, organized by CFIR domains. This figure illustrates how the research team anticipated and addressed key challenges to adolescent participation in human-centered design workshops by applying contextual adaptations mapped to the Consolidated Framework for Implementation Research (CFIR) domains. Each domain highlights engagement challenges (bolded) and corresponding facilitation strategies (bulleted) across the outer setting (community context), inner setting (school/implementation climate), individual (adolescent) characteristics, intervention characteristics, and process domains. Adaptations addressed hierarchical structures, cultural sensitivities, developmental considerations, engagement barriers, and limited familiarity with structured participatory methods. The framework illustrates how tailoring facilitation methods helped create a safer and more developmentally appropriate environment for meaningful adolescent engagement in intervention development.

Workshop planning and facilitation were conducted by a multidisciplinary research team with expertise in mental health, education, human-centered design, and adolescent engagement. The team included a psychiatrist (WFK), a psychologist with expertise in youth mobilization (PMA), a former schoolteacher (ARA), four graduate students (IA, MMMB, PKG, BA), and a pediatrician-researcher with prior experience applying human-centered design in resource-limited settings (CLL). All team members, except for one, were local Ghanaians, ensuring that workshop activities were contextually grounded in cultural and linguistic norms. Drawing from this diverse expertise, the team proactively identified potential engagement challenges and crafted structured activities to address them (Figure 2).

#### Workshop 1: Understanding Adolescent Perspectives on Mental Health (Inspiration Phase)

The first workshop aimed to explore adolescent perceptions of mental health, including perceived barriers, facilitators, and support systems. Prior formative research with school guidance counselors and community-based mental health leaders identified key engagement challenges that could hinder adolescent participation: (1) limited familiarity with mental health concepts, (2) potential discomfort discussing sensitive topics such as stigma and substance use, (3) developmental differences in cognitive processing and communication styles, and (4) the need to build trust and rapport with facilitators to encourage open dialogue.

**Figure 3:**
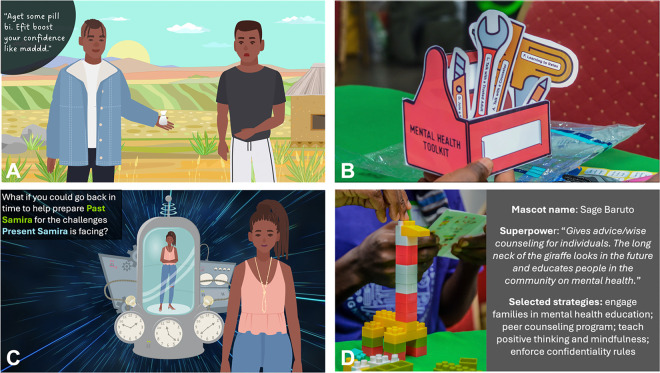
Visual Representation of Co-Design Activities Used to Engage Adolescents in School-based Mental Health Intervention Development. This figure presents key activities from the co-design workshops, illustrating how structured facilitation supported adolescent participation in developing school-based mental health interventions. (A) Case-Based Scenarios – Illustrated vignettes depicting relatable adolescent experiences with mental health challenges were designed to prompt discussion and contextualize abstract mental health concepts. (B) Mental Health Toolkit Activity – Hands-on exercise where participants selected evidence-based mental health strategies and assembled a “toolkit” for coping with stress and emotional challenges. (C) Time Travel Exercise – A structured reflection activity guiding participants to differentiate between prevention and intervention strategies by building Mental Health Toolkits available to a peer both before and after experiencing distress. (D) School Mascot Activity – Students worked in school-based pairs to design a mental health mascot representing their ideal school-based support strategies, incorporating key intervention components.

To address these challenges, the multidisciplinary research team designed the workshop to scaffold participation through interactive case-based scenarios, structured discussion techniques, and trust-building activities (Table 1). Case scenarios were developed based on findings from prior interviews to ensure realism and relatability. These scenarios illustrated common adolescent mental health challenges (e.g., peer pressure, substance use, academic stressors) and were visually depicted using adapted Adobe Stock character sets that were refined to reflect the local context (Figure 3A). Discussion prompts were embedded within the cases to structure conversations and support adolescents in articulating their perspectives. This interview guide was developed for this study using the Consolidated Framework for Implementation Research (CFIR).^34,35^ The interview guide and related materials used in this study are available as Supplementary Files 1 and 2.

Additional engagement strategies included an opening presentation that introduced the research team’s goals (“our goal is to learn from you and your experiences”), set expectations (“please speak honestly and freely”), and assured participants of confidentiality, explaining how responses would be compiled and de-identified. To foster rapport and encourage participation, the session included interactive icebreaker activities, such as movement-based games like charades. Then, the discussion opened with a collective group definition exercise, where adolescents collaboratively defined “mental health” and “mental wellness.” This activity helped establish a common conceptual foundation, allowing facilitators to assess prior knowledge levels and adapt discussion pace and complexity accordingly. Case-scenario discussions alternated between large-group and small-group formats, with gender-segregated sessions to create safer spaces for sharing culturally sensitive experiences.

#### Workshop 2: Co-Designing School-Based Mental Health Strategies (Ideation Phase)

The second workshop aimed to translate adolescent perspectives on mental health challenges into preferred strategies for school-based prevention interventions. Findings from Workshop 1 directly informed modifications to the second workshop’s design, particularly in addressing three key engagement challenges: (1) communicating the abstract concept of prevention, (2) guiding adolescents to focus on external, school-based strategies rather than individual willpower, and (3) sustaining engagement throughout the session following observations of attention fatigue in Workshop 1.

To address these challenges, the research team designed structured, choice-based activities to scaffold decision-making and encourage active participation (Table 1). Recognizing that adolescents may have limited prior exposure to mental health prevention strategies, the workshop was structured to introduce and present a menu of evidence-based strategies rather than relying on adolescents to generate ideas from scratch. This approach ensured that proposed interventions remained realistic, actionable, and within the scope of implementation.

For individual-level strategies, adolescents were asked to build a “Mental Health Toolkit,” selecting from printed tools labeled with different coping and prevention strategies such as “find a mentor,” or “talk to a counselor” (Figure 3B). To reinforce the concept of prevention, the team used time travel as an analogy to engage participants in creating Mental Health Toolkits for a peer experiencing severe stress – first in the present and then retrospectively before symptoms emerged (Figure 3C). This helped adolescents distinguish between reactive and proactive mental health interventions and clarified their preferences for intervention strategies.

For school-level interventions, adolescents participated in a robotics-type activity to build a School Mental Health Mascot. Working in pairs from the same school, students used plastic building blocks to create mascots that embodied their vision for a supportive school environment. Each pair assigned their mascot a mental health “superpower” and selected up to four intervention strategies from a menu of school-based preventative approaches. This structured activity fostered creativity while guiding students toward actionable and feasible solutions. It also served as a tangible demonstration of the adolescents’ grasp of mental health prevention concepts. To further sustain engagement and promote collaboration, the activity included a competitive component that leveraged school pride—identified in Workshop 1 as a key motivator.

Throughout the workshop, structured facilitation by the multidisciplinary research team ensured that activities remained accessible and engaging. Facilitators with backgrounds in psychology, education, and youth engagement played key roles in guiding discussions, encouraging participation, and ensuring perspectives of adolescents were accurately captured.

### Data Collection and Analysis

Data were collected from multiple sources: structured discussion notes, facilitator reflections, workshop artifacts (worksheets, written ideas), and audio recordings of large-group discussions. Audio recordings were not fully transcribed but were selectively reviewed for clarification. Notes captured discussions during both large and small group sessions and were recorded by multiple facilitators (MMB, CLL, PKG, IA).

Data were analyzed using thematic analysis. Because multiple facilitators recorded notes during all discussions, notes were entered into a comparative matrix to ensure consistency and completeness. Audio recordings were reviewed to resolve discrepancies or unclear notes. Two coders (MMMB, BA) independently identified themes, which were refined through whole-team discussions to resolve discrepancies and achieve consensus.

## RESULTS

A total of 24 adolescents (ages 16–23 years; 45% male, 55% female) from all twelve public senior high schools participated in the workshops. Results are organized by workshop phase, detailing the planning considerations, implementation strategies, and key insights generated from each session.

### Workshop 1: Adolescent Perspectives on Mental Health

Workshop 1 explored how adolescents conceptualized mental health, the barriers and facilitators they identified, and their perceptions of the role of schools in supporting student mental health. Initially, many students were hesitant to engage, likely due to unfamiliarity with facilitators and uncertainty about expectations. Establishing a collective definition of mental health fostered a shared conceptual understanding, which helped facilitators guide the discussion and informed refinements for Workshop 2. However, variability in conceptual clarity remained, as some students provided broad or tangential responses, suggesting differences in prior exposure to mental health concepts. Facilitators also observed that adolescents often framed mental health solutions from an individual lens, focusing on personal resilience to overcome challenges, rather than the role of systemic supports within schools and communities.

Case-based scenarios and cartoon visuals were effective in prompting discussion, particularly in helping adolescents relate abstract mental health concepts to real-world examples. Gender-segregated discussions successfully created a more comfortable environment for discussing culturally sensitive topics such as stigma and gender-based expectations. Engagement levels fluctuated, with higher participation in small-group settings and a decline towards the end of the session, likely due to attention fatigue. Facilitators observed that graduate student facilitators, who were closer in age to participants, played a key role in fostering rapport.

Workshop 1 highlighted key barriers and facilitators to adolescent mental health within schools and communities (Table 2). Adolescents described limited mental health awareness, stigma, and fear of judgment as major barriers to seeking support. Many felt their teachers lacked awareness of mental health and expressed concerns about confidentiality and student gossip. Cultural expectations shaped gender differences; male students discussed cultural norms around substance use modeled by community elders, while female students noted cultural gender roles that hindered open discussions of substance misuse. These insights guided refinements for Workshop 2, particularly in structuring activities to sustain engagement and shifting the focus from challenges to concrete intervention strategies. A detailed analysis of barriers and facilitators will be presented in a separate publication.

### Workshop 2: Co-Designing School-Based Mental Health Strategies

Compared to Workshop 1, adolescent engagement was higher, particularly during hands-on activities. The Mental Health Toolkit helped adolescents to identify and explore different strategies to address mental health challenges. The Time Travel Analogy enabled them to conceptualize mental health prevention in a concrete way, by distinguishing between proactive and reactive strategies and emphasizing the importance of addressing stress and emotional well-being before problems escalate. Top individual-level prevention strategies selected by students were mentorship, positive friendships, and mental wellness education.

The School Mental Health Mascot Activity was the most engaging and creative component. Working in school-based pairs, students designed and presented mascots representing their vision for a supportive school environment. Through this exercise, five priority school-based intervention strategies emerged: training and supporting teachers in mental health literacy, integrating mental health education into the curriculum, implementing mentorship programs, teaching positive thinking and mindfulness, and using entertainment-based methods for mental health education. The use of structured facilitation and a predefined menu of options helped guide decision-making while still allowing for creativity.

Observations from Workshop 2 reinforced the importance of structured facilitation in adolescent co-design. Guided activities helped sustain engagement and facilitated more concrete, actionable intervention ideas. While adolescents demonstrated enthusiasm for intervention development, discussions remained at a conceptual level, reinforcing the need for continued stakeholder co-design with multilevel stakeholders to refine and implement strategies. These findings informed the development of the next phase of work, including ongoing engagement through an adolescent advisory board.

### Post-Workshop: Formation of the Adolescent Advisory Board

Following Workshop 2, multiple students, parents, and teachers independently reached out to the research team, expressing a strong interest in continued engagement. Recognizing the value of sustaining adolescent input, the research team formed an Adolescent Advisory Board to ensure youth involvement in the next phases of intervention development. While this advisory board was not initially planned as part of the study design, its formation reflects adolescents’ desire for sustained participation beyond the workshops.

The Adolescent Advisory Board meets quarterly through a combination of WhatsApp-based discussions—chosen for accessibility and ongoing communication—and in-person meetings, promoting sustained and meaningful adolescent engagement. To integrate adolescents meaningfully into broader implementation efforts, two student representatives now participate in stakeholder co-design meetings with school and community partners. This integration ensures that youth perspectives directly inform ongoing intervention refinement and implementation strategies. For long-term sustainability, leadership is being transitioned from the research team to a local community-based organization. This approach helps ensure that adolescent engagement extends beyond the research period, embedding youth participation into local structures and fostering continued ownership of mental health interventions.

## DISCUSSION

This study adapted and implemented co-design methods to engage adolescents in developing school-based mental health interventions in northern Ghana. While co-design is increasingly recognized as a valuable strategy for intervention development, its application with adolescents—particularly in LMICs—requires intentional adaptations to ensure meaningful participation. First, our findings demonstrate how structured facilitation and choice-based engagement models supported adolescent participation, allowing them to articulate concrete intervention priorities. Second, our study illustrates how contextually adapted co-design methods addressed sociocultural and cognitive barriers, fostering engagement in a setting where participatory research methods are less familiar. Third, we found that structured engagement strategies helped adolescents shift their framing of mental health challenges – from viewing them solely as individual responsibilities to recognizing the need for broader school and community-level interventions and support.

### Contextualizing Co-Design in LMICs for Adolescent Mental Health

Co-design methods have traditionally been applied with adult stakeholders, where structured decision-making and implementation planning are more familiar.^7^ When applied to youth, studies often focus on what adolescents contribute rather than how their engagement is structured to ensure meaningful participation.^17^ Our findings contribute to this growing field by documenting specific adaptations that enhanced adolescent participation in an LMIC setting, where power hierarchies, cognitive developmental differences, and limited familiarity with participatory methods require intentional facilitation approaches.^36^

Our findings reinforce the importance of structured facilitation in adolescent co-design. Open-ended ideation alone may not be effective, particularly when working with youth who have limited prior exposure to participatory design methods, lower confidence in sharing ideas, or less familiarity with the range of possible intervention strategies. Instead, guided decision-making exercises, visual tools, and structured engagement techniques facilitated deeper participation and more actionable intervention ideas. For example, the Mental Health Toolkit and Time Travel Analogy allowed adolescents to conceptualize prevention strategies in a concrete, developmentally appropriate way. Prior research suggests that choice-based engagement models can enhance adolescent decision-making by reducing cognitive load and making abstract concepts more accessible.^37,38^ Our study extends these findings by demonstrating that structured facilitation not only sustains engagement but also deepens adolescent understanding of mental health as a shared responsibility.

Additionally, we found that establishing a shared conceptual foundation for mental health before co-design activities ensured alignment between facilitators and participants. The collective definition activity helped assess adolescents’ baseline understanding of mental health, reduce confusion, and tailor discussions accordingly. This step is critical in LMIC settings, where formal mental health education is often limited and where youth may have varied exposure to mental health concepts. By creating common ground, this strategy facilitated more focused, meaningful engagement throughout the co-design process.

### Unique Processes & Adaptations for the Ghanaian Sociocultural Context

Engaging adolescents in co-design within LMICs presents distinct sociocultural challenges, including hierarchical power structures, gender norms, and stigma surrounding mental health discussions.^36,39^ Our study highlights several key adaptations that enhanced adolescent participation in the specific context of northern Ghana. First, gender-segregated discussions provided safe spaces for discussing sensitive topics, allowing adolescents to express concerns more openly. Second, peer-driven engagement strategies—such as incorporating group work and graduate student facilitators—helped foster trust and relatability. Third, leveraging school identity through activities like the School Mental Health Mascot Activity tapped into existing community structures to sustain engagement and motivation. These adaptations align with broader research on youth engagement in participatory research in LMICs, suggesting that tailoring engagement strategies to local cultural norms enhances feasibility and effectiveness.^24^ However, few co-design studies in LMIC settings systematically document these cultural and structural adaptations, making replication challenging.^6,26^ This study demonstrates how facilitation strategies can be flexed to navigate sociocultural barriers and enhance youth participation. Moreover, our findings underscore the importance of multidisciplinary teams with local expertise, who are critical to identifying engagement barriers and co-creating contextually adapted solutions to foster meaningful and productive youth participation.

### Adolescent-Identified Mental Health Priorities & Conceptual Shifts

The structured co-design process enabled adolescents to identify and prioritize five key school-based preventative interventions: training and supporting teachers in mental health literacy, integrating mental health education into the curriculum, implementing mentorship programs, teaching positive thinking and mindfulness, and using entertainment-based methods for mental health education. The prioritization process, which guided adolescents through scaffolded decision-making activities, ensured that selected strategies were both feasible within the school context and aligned with their lived experiences. These priorities directly informed the next phase of intervention development, including plans for continued stakeholder engagement and the refinement of implementation strategies in collaboration with school and community leaders.

An important outcome of the workshops was that structured engagement influenced adolescent perceptions of mental health support. Initially, many participants viewed mental health challenges as individual struggles, emphasizing personal resilience and willpower as primary coping mechanisms. However, through structured engagement, adolescents reframed their understanding, shifting their focus toward the roles that schools and communities could play in supporting adolescent well-being. This shift is evident in their prioritized school-level interventions, which reflect a balance between individual-level strategies, such as mentorship and mindfulness education, and systemic school-level approaches, including curriculum integration and teacher training. The ability of co-design methods to shape adolescent perspectives is a novel contribution to the literature and highlights the potential of participatory engagement to shift mental health narratives in LMIC settings.

Additionally, the emergence of the Adolescent Advisory Board highlights the potential for sustained youth engagement beyond initial co-design workshops. While adolescent co-design often focuses on ideation, our findings suggest that structured engagement mechanisms can extend youth participation into implementation and advocacy. Future research should explore how to operationalize sustained adolescent involvement in intervention refinement and implementation within school and community mental health initiatives.

### Study Limitations

This study has several limitations. First, participants were nominated by their schools, likely selecting higher-performing students, which may have excluded lower-performing or marginalized adolescents’ perspectives. Second, the exclusive focus on in-school youth overlooks out-of-school adolescents, who may face even greater mental health challenges and service barriers. Third, the absence of systematic measurement of adolescent engagement levels limits deeper insights into potential variations by age, gender, or school type. Future research should address these gaps by ensuring broader representation and incorporating structured assessments of engagement.

## CONCLUSIONS

This study demonstrates how human-centered design can be adapted to enhance adolescent engagement in the development of school-based mental health interventions in LMICs. A key contribution is the adaptation of co-design to address multiple potential challenges in youth engagement in Ghana, creating a replicable approach for adolescent-driven intervention development that can be adapted across other LMIC settings and health topics. By integrating structured facilitation, peer-driven engagement, and culturally responsive participatory methods, this study advances implementation science approaches for co-design in resource-limited settings.

Future research should explore the long-term impact of adolescent engagement on intervention effectiveness, assessing whether structured youth participation influences implementation fidelity, scalability, and real-world adoption. Expanding co-design approaches to out-of-school youth and other marginalized populations will be essential for broader representation and inclusivity. To enhance feasibility in LMICs, future work should identify low-resource adaptations of structured facilitation methods, exploring how participatory approaches can be embedded within school and community-based mental health policies to promote sustainability at scale. Ultimately, advancing youth-inclusive implementation strategies has the potential to strengthen mental health systems and improve outcomes for adolescents by ensuring interventions are both contextually relevant and youth-centered.

## Figures and Tables

**Table 1.** Structured co-design activities, implementation details, and key adaptations of human-centered design workshops in Tamale, Ghana

Activity	Objective	HCD Phase	Implementation Details	Key adaptations and insights
Opening & icebreaker activities	Establish rapport, set expectations, and create a comfortable environment for discussion	**Inspiration**, Ideation	Format: Large-group interactive activities Time: 20 minutes Components: • Confidentiality agreement: set ground rules for privacy and respectful discussions • Interactive ice breakers e.g., friendship bingo where adolescents find peers with shared experiences or hobbies	Used movement-based activities to energize students and promote early participation Reinforced peer connections to reduce anxiety about discussing mental health Facilitators modeled participation to normalize engagement
Collective definition exercise	Establish a shared understanding of mental health and wellness	**Inspiration**	Format: Large group discussion Time: 10 minutes Example prompt: “What does mental health mean to you? What words come to mind?”	Used structured facilitation instead of open-ended brainstorming to guide responses Allowed peer validation to reinforce concepts Facilitators adjusted language based on observed comprehension levels
Case-based scenario discussions (Figure 3A)	Encourage adolescents to explore mental health challenges through relatable examples	**Inspiration**	Format: Large and small, gender-based small group discussions Time: 45 minutes per case Example prompt: “What are some reasons someone might feel like they can’t get help when they are feeling very sad or anxious?” Follow up: “Are there any cultural beliefs or attitudes within your schools/communities that might contribute?”	Developed cartoon-based visual scenarios to make abstract topics more accessible Used CFIR-guided prompts to structure discussions Included gender-segregated groups for discussion of sensitive topics
Mental Health Toolkit (Figure 3B)	Help adolescents identify and prioritize personal coping strategies	Ideation	Format: Individual tool selection and small group discussion Time: 30 minutes Example prompt: “Choose one tool that you would use to manage stress. Why?”	Used printed cards with labeled strategies instead of open-ended responses Provided structured choice categories (e.g., “talk with a counselor,” “art/music therapy,” “plan fun activities”) to scaffold decision-making
Time Travel Analogy (Figure 3C)	Illustrate the difference between intervention and prevention strategies	Ideation	Format: Large-group presentation of a case scenario featuring a student facing academic and life stressors, followed by small group discussions to create Mental Health Toolkits addressing the student’s challenges before and after stress onset Time: 40 minutes Example prompt: “If you could go back in time, what tools could help this student prepare for her future challenges?”	Used time travel as an analogy to make prevention more easily understandable Guided discussions toward structural solutions beyond personal resilience using structured choice categories
School Mental Health Mascot (Figure 3D)	Help adolescents identify and prioritize school-based prevention strategies	Ideation	Format: Paired activity (same school pairs) and large group presentation/competition Time: 60 minutes Example prompt: “Design a school mascot that represents a mentally healthy school. What features does it have?”	Provided a menu of school-based strategies for students to select up to 4 choices Created avenue for creative expression while keeping discussion structured Leveraged school pride as a motivator for engagement

**Table 2.** Adolescent-identified barriers, facilitators, and strategies for school-based prevention in Tamale, Ghana (n=24). Barriers, facilitators, and preferred mental health prevention strategies were identified by adolescents during human-centered design workshops. Data are categorized according to the Consolidated Framework for Implementation Research (CFIR) domains.

CFIR Domains	WORKSHOP 1 RESULTS: Facilitators (+) / barriers (−) to mental health	WORKSHOP 2 RESULTS: Selected implementation strategies (relevant activity)
**Individuals** *Adolescents*	Access to information and knowledge +/− Fear of judgment − Peer pressure and substance use −	Learn about mental wellness (Mental Health Toolkit) Make and keep positive friends (Mental Health Toolkit) Find a mentor (Mental Health Toolkit)
**Inner setting** *Tamale high schools*	Teacher/guidance counselor engagement +/− Available resources +/− Access to information and knowledge − Confidentiality and trust − Relative priority −	Train and support teachers (School Mascot) Teach positive thinking and mindfulness (School Mascot) Offer mentorship (School Mascot)
**Outer Setting** *Community and social context*	Stigma − Support from community leaders − Social determinants of health −	
**Intervention and Process**	Champions + Opinion leaders +/− Reflection evaluation −	Include mental health in the curriculum (School Mascot) Use entertainment for education (School Mascot)

## Data Availability

Deidentified survey data and interview transcripts are available upon reasonable request. Data can be accessed by contacting Claudia Leung at Claudia.Leung@bsd.uchicago.edu or via ORCID ID: 0000-0003-3389-9115. Data reuse is permitted for research purposes upon approval, in accordance with ethical guidelines and any applicable institutional or regulatory requirements. Additional supporting materials, including study protocols, co-design materials, and statistical analysis plans, may be available upon request.

## Abbreviations

AAB: Adolescent Advisory Board
AMPATH: Academic Model Providing Access to Healthcare
CFIR: Consolidated Framework for Implementation Research
LMIC: Low- and Middle-Income Countries
TOLECGH: Total Life Enhancement Centre Ghana
